# Open access publication in the fields of human fertility, infertility and early pregnancy

**DOI:** 10.1186/2054-7099-1-1

**Published:** 2015-04-21

**Authors:** Danny J Schust, Elizabeth S Ginsburg

**Affiliations:** 1 Department of Obstetrics, Gynecology and Women’s Health, University of Missouri School of Medicine, 500 North Keene Street, Suite 203, Columbia, MO 65201 USA; 2 Department of Obstetrics and Gynecology, Brigham and Women’s, Hospital, Harvard Medical School, 75 Francis Street, ASB1 3rd floor, Boston, MA 02115 USA

## Abstract

The study of human fertility has expanded dramatically since the birth of IVF and with it, the body of literature. That said, the availability of open access publication in the field remains somewhat limited. *Fertility Research and Practice* offers a new open access, peer-reviewed venue for quality clinical, basic science and translational studies and scholarly reviews in human fertility, infertility and early pregnancy. The journal specifically encourages works from international contributors and from investigators in less-resourced environments and has gathered an editorial board with a breadth of geographic representation to support this mission.

Together with Biomed Central, Drs. Elizabeth S. Ginsburg and Danny J. Schust are pleased to announce the launch of a new peer reviewed open-access online journal entitled *Fertility Research and Practice*. We hope that readers enjoy this new, relevant addition to the medical literature and welcome submissions.

In an era notable for rapid expansion in the choices an investigator, clinician and author has available for publication of a particular manuscript, it is important to distinguish a new entry by its unique characteristics, and *Fertility Research and Practice* has many. We understand that the experiences and interests of basic science and clinical researchers, medical and paramedical practitioners and thought leaders from all regions of the world have the potential to instruct and impact healthcare locally and more broadly. We therefore encourage a wide diversity of submissions.

The scope of *Fertility Research and Practice* includes male and female fertility and early pregnancy. While our focus is on human work, we appreciate that much of what we know about humans was first learned through the study of animal models and/or through in vitro modeling, so we also welcome experimental work with translational applications. We welcome basic science, translational, clinical and epidemiological studies that may be presented as traditional data-based manuscripts, brief reports, reviews and commentaries, case reports and case series. *Fertility Research and Practice* also has the capability to accept video submissions. Our interests in fertility and early pregnancy are also broad, diverse and inclusive. Among our many areas of interest are the diagnosis and correction of endometriosis and congenital and acquired anomalies of the male and female reproductive tracts. We have additional interests in reproductive infectious diseases, male and female fertility diagnostics (including imaging) and assisted reproductive techniques, including embryology. Unlike some fertility journals, our scope has a specific emphasis on early pregnancy, including implantation, and diagnosis and treatment of pregnancies of unknown location and sporadic and recurrent early pregnancy loss. We have a particular interest in the effects of environmental exposures on fertility and early pregnancy and on epidemiology and welcome novel and cutting-edge diagnostic and therapeutic approaches. Drs. Ginsburg and Schust have been fortunate to have assembled a very skilled and respected group of international scholars on their editorial board whose expertise covers the breadth and depth of *Fertility Research and Practice’s* scope of interest.

First and foremost, we are an open access journal whose mission includes wide distribution without geographic or cost barriers. As an open access journal, authors are requested to contribute to publication costs, an unavoidable mandate of the open access business model that is rapidly expanding into non-open access journals as well. To satisfy this financial business mandate while encouraging the greatest possible geographic and economic diversity among submitted and published manuscripts, however, BioMed Central has a policy of reducing or waiving publication fees for submissions from less developed/less resourced authors and institutions. This integral part of their business plan makes publication in a BioMed Central Journal such as Fertility Research and Practice an option for all authors and from institutions of all sizes.

BioMed Central has an illustrious history in the open-access publication field and is committed to rapid and supportive review (usually in less than 14 business days; if not an explanation of the delay is forwarded), widespread distribution not limited by mail access or library subscription, and access to readers without cost. Open access publication benefits authors by increasing access and readership when compared to subscription-based publication [1], which enables the potential for increased citations [2, 3]. Open access articles also fulfil the mandates of most funding agencies for distribution of research findings. Attributes of the open access model should not be tempered by a reduction in the quality of the published manuscripts; this is assured within *Fertility Research and Practice* by the demanding standards of BioMed Central [4], the expertise, training and experience of the journal’s editorial board and editors-in-chief and the journals inclusion in the CROWN Initiative. The Core Outcomes in Women’s Health (CROWN) initiative is led by an international group of journal editors who are making a concerted effort to reduce the heretofore widespread inconsistencies in the reporting of outcomes in women’s health research. Such inconsistencies make studies of a particular intervention or its outcomes difficult to compare and therefore hinders our ability to make recommendations based on the literature. We at *Fertility Research and Practice* are committed to reducing such inconsistencies in our own published articles and will help the initiative in creating recommendations when they apply to our scope.

In the inaugural edition of *Fertility Research and Practice*, we present four articles that exemplify the breadth of our scope of interest. Two of the inaugural articles address surgical issues within reproductive medicine. Brady and Styer [5] provide a case report and literature review surrounding a vexing patient with recurrent idiopathic ovarian torsion while Bailey, et al. [6] report on an animal model that uses omental flaps to aid in adhesion prevention after abdominal surgery. The third manuscript in the inaugural issue is epidemiology-based and illustrates the journal’s international interests. In this manuscript, Rabbi and Kabir [7] assess changes in birth intervals and mean age of childbearing in Bangladesh. Our final inaugural manuscript [8] reports on a clinical study of time to pregnancy in patients with recurrent pregnancy loss stratified by spontaneous versus assisted conception.

Please enjoy the launch edition of *Fertility Research and Practice*. We welcome your submissions.
